# Insights into a 429-million-year-old compound eye

**DOI:** 10.1038/s41598-020-69219-0

**Published:** 2020-08-13

**Authors:** Brigitte Schoenemann, Euan N. K. Clarkson

**Affiliations:** 1 Zoology Department (Neurobiology/Animal Physiology and Biology Education), University of Cologne, Herbert-Lewin-Straße 10, 50931 Cologne, Germany; 2 Grant Institute, School of Geosciences, University of Edinburgh, West Mains Road, Edinburgh, EH9 3JW UK

**Keywords:** Evolution, Neuroscience, Physiology, Zoology, Ecology, Ocean sciences, Optics and photonics

## Abstract

In all arthropods the plesiomorphic (ancestral character state) kind of visual system commonly is considered to be the compound eye. Here we are able to show the excellently preserved internal structures of the compound eye of a 429 Mya old Silurian trilobite, *Aulacopleura koninckii* (Barrande, 1846). It shows the characteristic elements of a modern apposition eye, consisting of 8 (visible) receptor cells, a rhabdom, a thick lens, screening pigment (cells), and in contrast to a modern type, putatively just a very thin crystalline cone. Functionally the latter underlines the idea of a primarily calcitic character of the lens because of its high refractive properties. Perhaps the trilobite was translucent. We show that this Palaeozoic trilobite in principle was equipped with a fully modern type of visual system, a compound eye comparable to that of living bees, dragonflies and many diurnal crustaceans. It is an example of excellent preservation, and we hope that this manuscript will be a starting point for more research work on fossil evidence, and to develop a deeper understanding of the evolution of vision.

## Introduction

Trilobites are extinct marine arthropods that dominated the ecosystems of the Palaeozoic. From the very beginning of their appearance they were equipped with compound eyes, which during the Cambrian explosion and later differentiated into highly diverse visual systems. A well-known trilobite is the small *Aulacopleura koninckii* (Barrande, 1846^1^), occurring in great numbers within a layer of mudstone of 1.4 m thickness on Na Černidlech Hill and Špičatý Hill near Loděnice in the Czech Republic (Silurian, Wenlock, Liten Formation, Motol Member, *Monograptus flexilis*-Zone). Sedimentary evidence from the mudstone beds, probably deposited over a period of just a few thousand years, together with the mode of occurrence of the biota itself, suggest that *A. koninckii* lived in an environment of fluctuating oxygen availabilty, and its 'olenimorph' morphology commonly is associated with oxygen-poor settings^2,3^. The French-Czech paleontologist Joachim Barrande, a pioneer of trilobite research, was the first to have excavated this location and he described the trilobite as *Arethusina konincki* in 1846. The name *Arethusina*, however, had been used already for a foraminiferal protist, and Hawle and Corda suggested the name *Aulacopleura* in 1847^4^.

**Figure 1:**
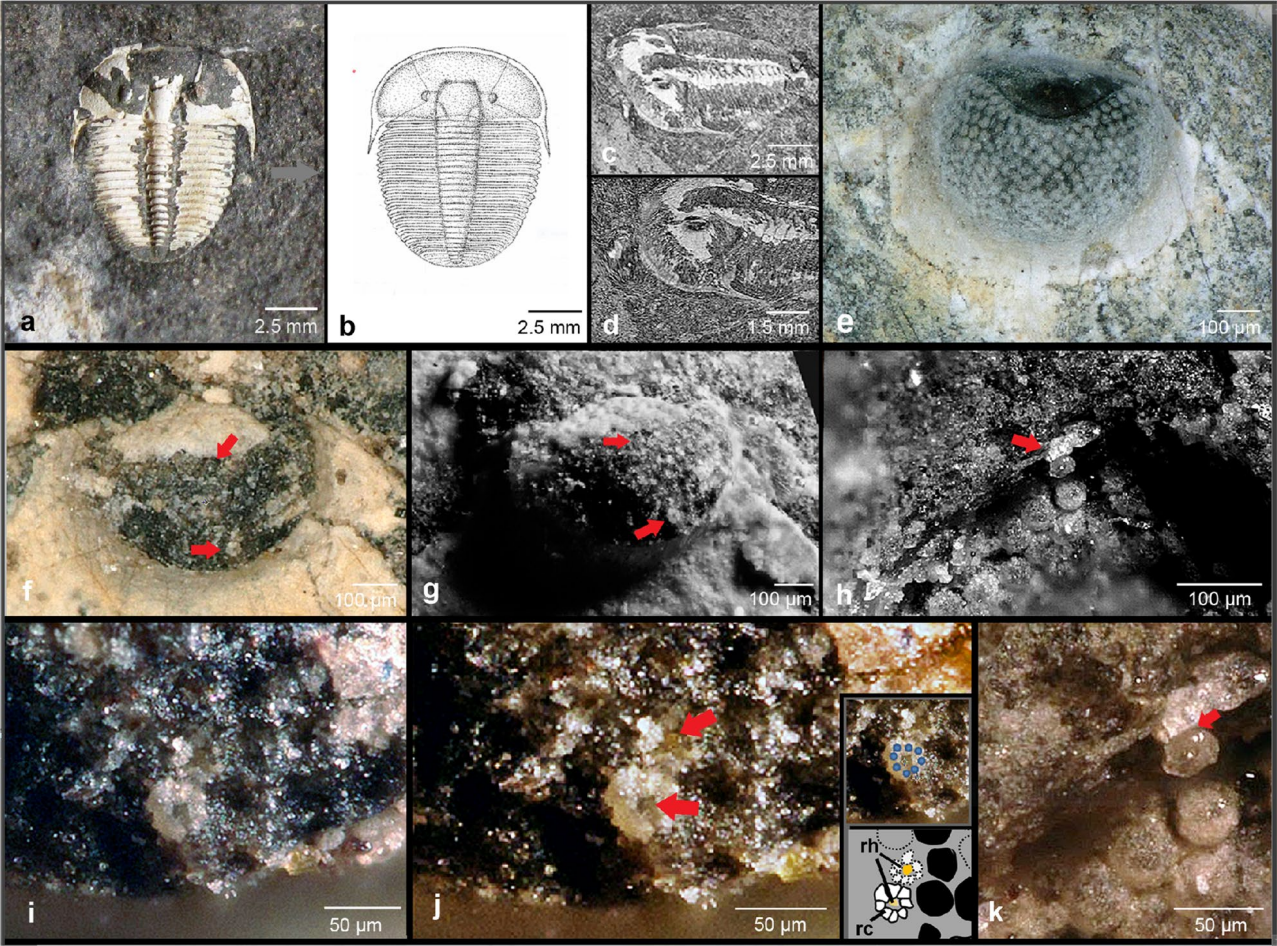
The apposition compound eye of *Aulacopleura koninckii* (Barrande, 1846^1^). (**a**) *Aulacopleura koninckii* (Barrande, 1846), specimen investigated here (Špičatý Hill Loděnice, Motol Fm., *Cyrtograptus lundgreni*-Zone, Silurian, Wenlock, Homerian. (**b**) Drawing of *A. koninckii.* (**c**) Oblique surface rendition. (**d**) Oblique view of the cephalon. (**e**) Left eye of an intact specimen. (**f**) Overview of the investigated eye, arrows indicate here illustrated visual units (i,j, Fig. 2a). (**g**) The same as (f), the enhanced contrasts here clearly show the empty cavities as left by the fallen-out visual units. Note the flaky white relics of decayed units still residing in the cavities. (**h**) Individual visual unit. (**i**) Rosette formed by the fossilised relics of the receptor cells surrounded by empty cavities. (**j**) Rosette formed by the fossilised relics of the receptor cells, note that the upper adjacent elements also shows a rhabdom very clearly (red arrow). The central rhabdom shows up several subunits. Inserts: Position of the relics of receptor cells; schematic drawing of **(j)**. (**k**) Fossilised relic of a complete visual unit with lens, and putatively a thin crystalline cone (red arrow).

**Figure 2:**
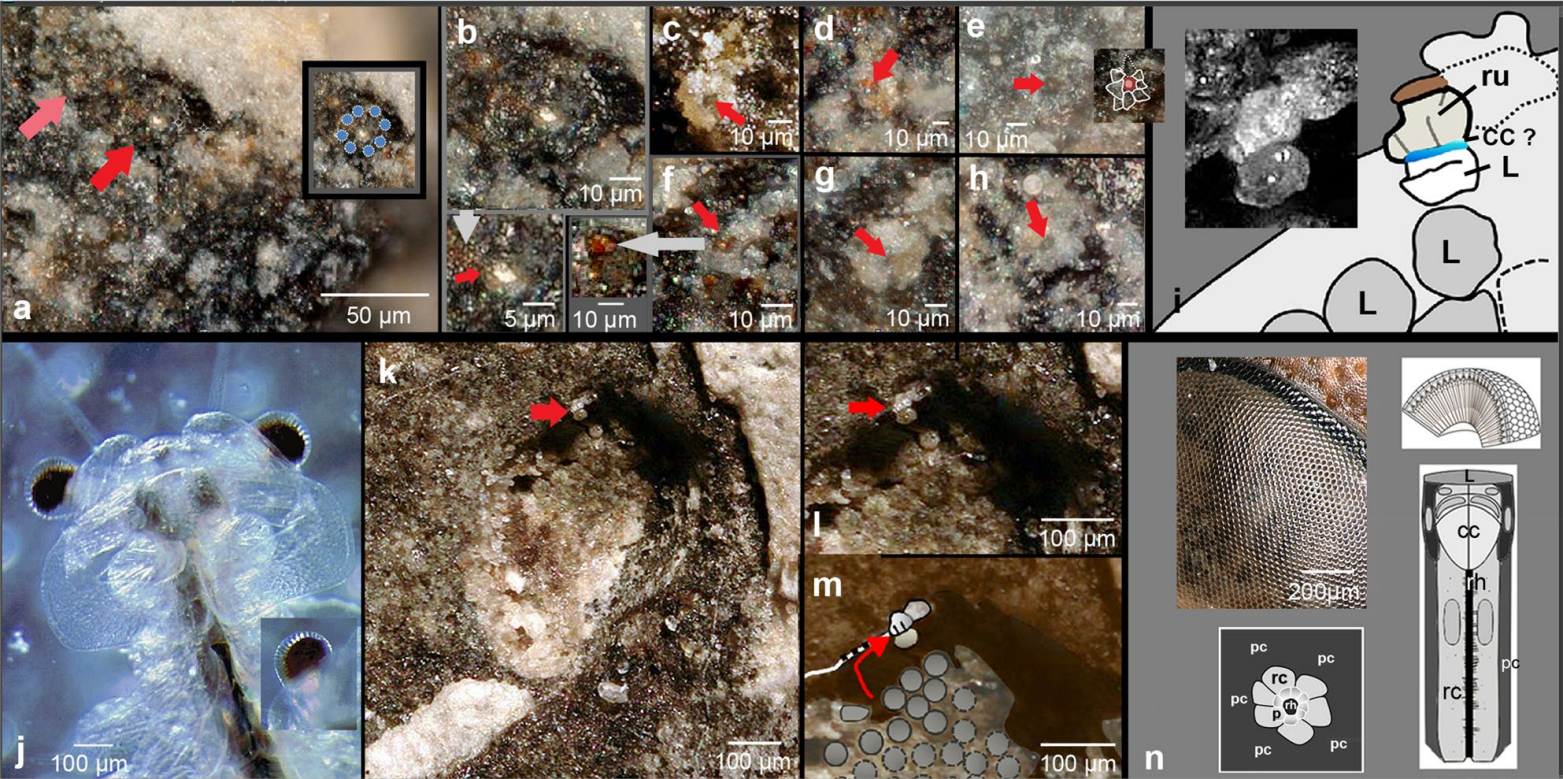
Ommatidial structures and their interpretations. (**a**) Overview of another sensory unit, fossilised in a different way (red arrow); another, slightly weathered adjacent (pink arrow). Insert: Position of the relics of receptor cells marked. (**b**) (a) in detail. Note the rhabdom embraced by spherical elements, interpreted as putative ‘palisades’^63–65^. (**c–h**) More examples of receptor-cell-rosettes with rhabdoms (red arrows) surrounded by palisades, f repeats the spherical elements very clearly. (**i**) Schematic drawing of (Fig. 1k), the putative thin crystalline cone in blue. (**j**) Translucent head of *Artemia salina* (Linnaeus, 1758), showing up the screening pigments inside of the compound eye. (**k**) Overview of the counterpart of the eye, red arrow indicates the position of the individual visual unit of Figs. 1k, 2i. (**l,m**) Illustration of how the isolated visual unit may have come up to its position, being stripped of the layer of lenses seen at their proximate surface below. (**n**) Modern compound eye of a hornet (*Vespa crabro Linnaeus*, 1758) and schematic drawing of an apposition compound eye, ommatidium and its cross-section. *c* cavity, formerly containing the receptor unit, *cc* crystalline cone, *p* palisade, *pc* pigment cells, *rc* receptor cell, *rh* rhabdom, *ru* receptor unit, *L* lens; **o–q, s** from the right eye.

*A. koninckii* (Figs. 1a–d, 2j) is a morphologically conservative trilobite^5^. It has been the subject of many recent studies, particularly of growth and ontogenetic development^6,7^. To obtain insight into sensory structures, such as those of compound eyes, is another challenge for different reasons. For a long time it has been thought to be most unlikely that soft tissues, such as neural tissues or even receptor cells could be preserved in the fossil record. Special modes of preservation, such as phosphatisation, or those of so-called Lagerstätten sensu Seilacher^8,9^ are needed. In such Lagerstätten carcasses were buried under anoxic conditions and with a low presence of bacteria, thus hardly any decomposition took place, and both—major as well as fine biological features—became preserved. Examples of Lagerstätten are the Maotianshan Shales and Burgess Shales of the Cambrian, the Devonian Hunsrück Shales, the Carboniferous Mazon Creek, or the Eocene Green River Formation, but there are many others with different compositions of their sediments, faunas and floras. Furthermore, to find tissue relicts at a cellular level, for the enclosing sediments a particle size smaller than the size of cells is necessary. Thus it is very uncommon to find fossilised individuals that retain internal structures of any former sensory system. Some records analysing nervous systems, such as brains^10–13^ and visual systems on receptor-cell-level, however, have been published^14–19^.

Recently, the eyes of a very early trilobite were described from the Lower Cambrian (base of the Atdabanian) of Estonia; this species *Schmidtiellus reetae* (Bergström, 1973)^20^ is amongst the oldest trilobites of all^18^. First known only as trace fossils at the stratigraphical level from which it comes, trilobite fragments then were found, and finally an almost complete, well preserved specimen was discovered. It is probably 'physically' (in body preservation) one of the oldest trilobites that will ever be found. Being excellently preserved in phosphate, it shows distinctive ommatidia. In some ways this is simpler than the eyes of modern apposition type^21,22^, yet the structures are clearly ommatidia. This eye shows a columnar ommatidium with ~ 8 receptor cells, and a central rhabdom. A whole series of ommatidia can be observed, and each of them is situated in a kind of cellular 'basket', separating the individual systems from each other, unlike typical modern apposition eyes. Another difference is that no clear lens can be distinguished, probably because the lens-generating part of the cuticle still had not originated in this early very thin shelled and fragile trilobite. Other points are pigment cells which are evidently not yet defined, and possibly there existed an elongated, pyramidally-shaped crystalline cone, although its shape is not very distinct. Nevertheless *S. reetae* possessed a typical apposition eye, even if at a quite primordial state^18^.

*Aulacopleura koninckii* (Barrande, 1846^1^) is a rather flat trilobite, usually not more than a millimeter or two in height. The preserved shell is very thin. It is no more than a few micrometers in thickness, often is stripped off, and normally just the internal mould (Steinkern) remains. Thus only the standard, typical features of the fossil arthropod are normally preserved. The eyes, protruding dorsally from the cephalon as small semi-ovals are particularly susceptible to damage, and are frequently broken off. The specimen documented here consists of part and counterpart, and the shell is partly preserved (Fig. 1b–d). In an exceptionally good, and possibly unique way it presents the internal structures of this compound eye in the Silurian trilobite. Partly it confirms what is known of the ocular anatomy of the Cambrian system of *S. reetae*^18^, but also offers new insights into vision in trilobite eyes.

## Apposition compound eyes and trilobites

The compound eyes, a plesiomorphic character of all arthropods^23^ during the last half billion years developed a high diversity of adaptations, in response to many different ecological constraints and opportunities. The most basic type, and still very common among especially diurnal arthropods, is the so-called apposition compound eye^21,22,24,25^.

Such eyes are today mainly represented among diurnal insects and crustaceans. They consist of up to 30,000 individual, more or less identical receptor units, so-called ommatidia (Fig. 2n), optically isolated from each other by a set of screening pigment cells. In terrestrial systems the cuticle forms a distinct lens, and a cellular crystalline cone directly below allows space for focusing the light onto a light guiding structure, the rhabdom. The latter is part of the receptor cells, and contains the visual pigments. The energy of the incident light changes their sterical configuration, which finally causes the generation of an electrical signal, led by the optical nerve to the central nervous system of the arthropod for further processing^26^.

In aquatic systems the difference of optical densities of chitin, the material of the cuticle, and water is not high enough for a cuticular lens to refract the light effectively. Here the functional part of the dioptric apparatus often consists mainly of the crystalline cone, forming an index gradient lens. All contrasts and colours inside the visual field of the ommatidium are focused onto the rhabdom, and in total over the entire compound eye it results a mosaic-like vision as first described by Müller in 1826^27^, and consequently by Exner^28^. Their acuity depends, among other factors on the number of facets, and the acceptance angle of the ommatidia representing the fineness of scanning of the environment. In more advanced systems the receptor cells may share functions such as the possession of sensitivity for different wavelengths, and adaptations to lower light intensities. Such elegant advancements as different kinds of superposition eyes, however, probably did not develop before the Devonian (419.2–358.9 Mya)^29^.

It is only in trilobites that the lens usually consisted (mainly) of calcite—a highly refractive material^30–36^. Especially if the lens is spherical, then it would have a short focal length. This would determine a short crystalline cone to give space for focusing the light onto the rhabdom. Thus, if *A. konickii* has a flat crystalline cone, this would be a strong indication that the lens genuinely contained calcite to a high degree. The taxonomic placement of trilobites has been much debated. Although they have been assigned to chelicerates^37^ there is a general consensus, based on many morphological characters that they belong to the group of Mandibulata^38,39^. By contrast with mandibulates, however, trilobites, seemed not possess crystalline cones. Very recently, however, the existence of crystalline cones has been suggested for trilobites^18,40^, but the evidence is far from unambiguous.

There are other organisms that use calcitic lenses, such as brittle stars or chitons e.g.^41–43^, but these 'eyes' function more or less just as light detectors. It has been challenged recently that trilobite lenses were formed of primary calcite^44^, but any clear evidence that it is not so, is missing so far. The trilobites are thus, as far as is known, the only arthropods to use calcite in lenses for image-forming vision, and a short (or even missing) crystalline cone would be a strong evidence that their lenses were to a high degree of calcite with its high refractive power.

### The discovery of sensory units (ommatidia) in the compound eye of *Aulacopleura koninckii* (Barrande, 1846)

Whereas the right eye of the specimen *Aulacopleura koninckii* (Barrande, 1846)^1^ investigated here is broken off, the left is still present. The visual surface sits on a low socle, and the visual surface towards the top is covered by a kind of lid, a so-called palpebral lobe, shading the light from above, and stabilising the eye itself. The spherically curved former visual surface shows impressions (Ø ~ 35 µm) of where the former visual units were positioned (Fig. 1e–g). They are packed in a regular, more or less squared to irregular arrangement (Figs. 1e,g; 2a). In the aquatic habitat where light is absorbed more easily than it is in air, the relatively small visual units indicate a life-style in a well-illuminated environment. Under good light conditions the diameter of ommatidial lenses can be smaller than under those of dim light, simply because under bright light conditions more photons per steradiant can enter the system to make it work efficiently. Consequently, enlarged facets will enhance sensitivity. So, to capture enough photons for threshold vision^45,46^ under bright light conditions the lenses can be of comparably smaller diameter. This enables the apposition-eye-system to install more lenses in the limited space of a compound eye, enhancing resolution. In the sea just in shallow water these bright light conditions are possible, so we just here will find small facets—or, the other way round, if we find small facets in a marine arthropod, it will not have lived under mid- or deep-sea-conditions.

The lens-diameters found here (~ 35 µm) are comparable to the facet dimensions of the shallow water branchiopod crustacean *Artemia salina* (~ 20µm^47^, Fig. 2j), the waterflea *Daphnia* (~ 35.5 + 2.1µm^48^), the shore crabs *Leptograpsus* (22 < *D* < 50 μm^49,50^) and *Uca lactea annulipes* (20 < *D* < 30 μm^51^). Many crustaceans living today possess superposition eyes, where the light from many facets is exploited by each individual rhabdom. Here the lens diameters are of comparable size: modern shrimps, living in tidally influenced coastal ecosystems, for examples in light-flooded seagrass beds (*Palaemonetes purgio* 30 µm^52^, *Acetes sibogae* 30µm^53–55^). Lobsters, crayfish and langoustines with superposition eyes, living at greater depth, show lens diameters of around 50µm^56–59^. Thus, if the facet diameter of *A. koninckii* is equal or even slightly smaller than those, which have systems of mechanisms of intensified light-capturing, one may establish that *A. koninckii* rather probably lived in well-lit conditions, and was not crepuscular or even nocturnal. The facets of apposition eyes of crustaceans living at greater depth are much larger (the facet diameter off the midwater amphipod crustacean *Phronima* (dorsal eye) is 130µm^58,60^, of the deep-sea isopod *Cirolana* ~ 150µm^61^, and of *Limulus* (Xiphosura, active at night), ~ 180µm^62^, all apposition eyes.

While in the trilobite investigated here most of the visual units fell out, or persisted just as relics of decayed material, some of the cavities in the eye described here still are filled with the remains of primary structures well preserved (Figs. 1f–k, 2a–I, k–m). Clearly a circular arrangement of 8 round elements (showing as white, Ø ~ 10 µm each) forming a kind of rosette (Ø ~ 35 µm) can be distinguished, grouped around a central component (showing as yellow, Ø ~ 8 µm, Figs. 1f,g; 2a–h). Protruding from the level of the surface and examined in side view, one can see that these elements are tops of small columns. Because this configuration sits in the position of the hollow pattern mentioned before, it must be interpreted as a relic of the visual unit still retaining its original position. The elements consequently are the remains of the receptor cells, the central structure represents the relic of the rhabdom (Figs. 1i,j; 2a–h). In two cases, (Fig. 2b,f) even grainy structures accompanying the rhabdom can be observed clearly, in others they are indicated as a grey collar around the rhabdom (Fig. 2c–e,g,h). Very cautiously they may be interpreted as relicts of so-called palisades ('Schaltzone' in old literature), a region of lower refractive index around the rhabdom, improving the properties of the rhabdomes as a light guide^63–65^.

Screening pigment cells belong to the 'standard equipment' of ommatidia (apposition type) to isolate the individual receptive units against each other optically. In trilobites the facets are separated against each other by 'cuticular walls', the interlensar scleras, and any screening pigment would not be necessary—obligate perhaps, however, due to the general phylogenetic context. In the oldest system of apposition eyes in trilobites, in *S. reetae*^18^, pigment cells are not visible. Indeed around the ommatidium of *A. koninckii* in Fig. 2a–h there is a dark ring without discernable cell structures around the receptor cells. Keeping in mind, however, that melanin^66^ and other screening pigments are very stable over millions of years^66–70^, we may be confronted here with relics of the former pigment screen. To establish such a melanin containing cell-girdle around the receptor cells is physiologically expensive. Its existence may indicate that the cuticle, thus the cuticular ‘walls’ which in trilobites isolate the ommatidia against each other (interlensar sclerae), and probably the whole cuticle of this trilobite was translucent, like in modern shrimps and other small aquatic crustaceans for example (Fig. 2j)—a perfect camouflage in water to be invisible. Fossil pigment cells as suggested here have been found so far for example in eurypterids^19^, fish^12^ and fossil insects^15,44^. It would be the first report of pigment in the apposition eye of trilobites.

The investigation of the counterpart revealed the lenses (Figs. 1k; 2k–m), which originally covered the visual units. The cavities seen in the part had formed post-mortally after the lenses were lost. Secondly, and most strikingly, there is actually a unique example of a complete visual unit residing within the membrane in the counterpart (Figs. 1k; 2k–m). It probably was stripped off from the rest of the lens-bearing part of the visual surface, seen from the proximate side in Fig. 2l,m. The almost spherical sensory unit (showing as white) is still covered by the lens and gives a unique impression of what this more than 400 million-year-old visual unit looked like. Between the lens and the receptor unit there is an indication of a very small band, just few micrometers wide. In this extremely flat 'girdle' no internal differentiations can be made out (no cone cells for example). To find them fossilized in the dimensions we discuss here, however, surely would be over-ambitious. Even though it seems that we may find here putatively traces of a small structure between lens and receptor unit, which in all other mandibulates is a crystalline cone (Fig. 1k red arrow; Fig. 2i blue band).

## Discussion

The visual system of the excellently preserved Silurian trilobite *Aulacopleura koninckii* (Barrande, 1846) is revealed as a classical apposition compound eye. It consists of eight receptor cells, grouped around a central element, the rhabdom. The rhabdom is whole, surrounded by small spherical elements (Fig. 2a,b,f), which may be the relics of so-called palisades, enhancing the light-guiding properties of the rhabdom^63–65^.

There is no interspace between the rhabdomeres, as could be typical for neural superposition eyes. If the rhabdom was divided into separated sub-elements (rhabdomeres) this would indicate a neuronal superposition eye. Here rhabdomeres of adjacent ommatidia oriented in the same direction are combined to neuronal cartridges, enhancing the field of light capturing, as we know, for example in the neural superposition eyes of dipteran flies^71–73^, and there are strong indications also that comparable systems occur in certain beetles, craneflies, earwigs and waterbugs^74,75^. It seems to be more probable, however, that the rhabdom here is uniform. Neural and optical superposition eyes do not appear until higher in the fossil record. Each of the receptor units in *A. koninckii* is topped by a thick lens, below which a small interface may be made out, perhaps indication of a (reduced?) crystalline cone. The system likely is embraced by a pigment screen. In summary, the visual system of this trilobite is surely an apposition compound eye, as typical of many diurnal crustaceans and insects of today.

As mentioned previously an even older compound eye of a trilobite has been described recently, that of the olenellid *Schmidtiellus reetae* Bergström, 1973^18^ from the base of the lower Cambrian^18^. In this trilobite the ommatidia lay in separated cellular 'baskets'. Here in *A. koninckii* the visual units still also lie very much separated from each other, though no cellular 'basket' can be discerned. If it existed it may have been very thin. In *A. koninckii* traces of pigment cells can be observed. At a first glance, they were not needed, because the optical isolation of the visual units in trilobites could be guaranteed by the compartmentalising cuticular 'walls', the so-called interlensar sclera^36^. If this optical isolation indeed were fully functional, the existence of the pigment girdle was a phylogenetic heritage. The physiological expensive establishment of a screening pigment system indicates, however, that the small trilobite *A. koninkii* likely was a translucent trilobite, comparable to modern shrimps and other smaller aquatic crustaceans with translucent shells (Fig. 2j), providing an excellent camouflage in water. This separation of the individual ommatidia by deep cuticular partitions, and the pigment screen protruding up to the lens (Fig. 2a,b), argue against an optical superposition eye as existing typically in shrimps and other modern decapod crustaceans. Here the light from many facets is exploited by each individual rhabdom, thereby enhancing photon capturing. The essential clear zone in the superposition compound eye, is absent in *A. koninckii.* Thus, the visual organs of this trilobite cannot be interpreted as an optical superposition eye.

An interesting point is the potentially existence of a crystalline cone, putatively represented as a very thin (~ 5 µm) layer, from outside appearing as a thin brownish collar between the lens and the adjacent receptor units (Fi Fig. 1k red arrow; Fig. 2i blue band). In well-known material from the fossil record are inverse relations. While in *A. koninckii* we find a thick lens, and putatively a thin crystalline cone, in the Jurassic crustacean *Dololcaris ingens* van Straelen, 1923^76^ we found, conversely, a thin lens and an elongated crystalline cone, as typical of many modern crustaceans^17^. In crustaceans often this elongated crystalline cone acts as an index gradient lens with high refractive properties, because the cuticular lens, due to its relatively low refractive index (chitin: n ~ 1.53, sea water: n ~ 1.334) cannot focus the light efficiently enough. If the crystalline cone is very thin, or even missing, it may be argued that the lens of *A. koninckii* is the focusing, light refracting element. The high refractive power of the system in *A. koninckii* is facilitated by the shape of the lens, which is approximately spherical. There is no question, in our view that trilobite lenses consisted to a high degree of calcite, which with its highly refractive power, optimised the system very efficiently, and a short or no crystalline cone were satisfactory. Finally, phylogenetically trilobites stay in the context to euarthropods, thus should possess crystalline cones, and the thin layer in this specimen between lens and receptor unit may be a relic of a thin, reduced crystalline cone, because of its relative position to the other elements of the ommatidium.

It is also quite remarkable that in the compound eye of *A. koninckii* the receptive unit is quite short (~ 30 µm). While for example in a bee the relation of the diameter of the ommatidium to the length of the receptive unit (length of the sensory cells) is ~ 1: 16 (aperture 20 µm, receptor length 320 µm^26^, p. 66), in *A. koninckii* it is ~ 1 : 1 (~ 30 µm Ø upper part of the receptor unit, ~ 30 µm length of the receptor unit, Fig. 1h,i). A long rhabdom in the centre of the receptor cells enables light to be absorbed over a substantial distance, and this is not the case in *A. koninckii*. This may be compensated by a relatively wide rhabdom (~ 8 µm), modern rhabdoms of diurnal arthropods typically have a width of 1.5–3.5 µm^26^, p. 110. In the eyes of nocturnal arthropods, and in the short ommatidia present in small eyes, often the width of the rhabdoms is enlarged. This normally facilitates to increase the angle over which photons are captured, but at the expense of resolution^77^, p. 70. Thus, this discovery of quite wide rhabdoms accords well with observations of recent compound eyes adapted to efficient photon capturing, as in nocturnal insects (8 µm^78–80^), or in mesopelagic crustaceans adapted to greater depths (8 µm^81^). Rhabdoms, however, may be even larger, as evident, for the cosmopolitan amphipod *Streetsia*, living at depths of 20–3000 m. Their apposition compound eyes have rhabdoms of 18–20 µm width^82^. These are just some examples. The small lens diameter (Ø ~ 30 µm) supports the interpretation, as given before that *A. koninckii* was a diurnally active arthropod, living in well-lit environments. Being not equipped with superposition eyes as for example modern shrimps, as discussed before the relatively wide rhabdom may have been of advantage to capture photons more efficiently. The 'wasted space' between the lenses, where wider lenses could have been installed, indicates that the need to capture as many photons as possible was not the reason for this design of the compound eye—regarding this, the similarity to the eye of *S. reetae*, however, indicates that the stout ommatidium with a wide rhabdom, separated from its neighbours, is a primordial character. One may mention, however, that just four million years after the appearance of *S. reetae*, trilobites with densely packed facets were clearly present (*Holmia*^18^), and that trilobites with crustacean-like crystalline cones and numerous, very flat lenses^17^ are likewise known.

## Conclusion

In conclusion we describe here, in the Silurian trilobite *Aulacopleura koninckii* (Barrande, 1846)^1^, from the Na Černidlech Hill and Špičatý Hill near Loděnice in the Czech Republic (Silurian, Wenlock, Liten Formation, Motol Member, *Monograptus flexilis*-Zone) well preserved the sensory structures of its compound eye. The analysis reveals that this trilobite had an apposition compound eye, each ommatidium consisting of a thick lens, a flat crystalline cone, and 8 (visible) receptor cells grouped around a quite wide rhabdom (~ 8 µm). Elements grouped around the rhabdom, putatively relics of palisades, may have enhanced the properties of the the rhabdom as a light guide. The receptor cells are encompassed by a pigmented system, probably pigment cells, the first reported for trilobites so far. The lens diameter suggests that *A. koninckii* lived under good light conditions, thus inhabited clear shallow waters, and probably was diurnal. In comparison to many modern diurnal arthropods, however, the ommatidial capsule appears short and stout. The relatively large interspaces between the ommatidia (~ 10–20 µm), thus wasting potential for capturing photons, may indicate a phylogenetic legacy. The latter view is supported by the similarity of the much older system of the trilobite *S. reetae*^18^. Finally the flat rather than elongated supposed crystalline cone may indicate that the lens itself had a high refractive power, in line with the understanding that it consisted of primary calcite.

Thus, the Silurian trilobite *Aulacopleura koninckii* (Barrande, 1846^1^) from the Czech Republic in principle had a typical apposition compound eye, comparable to that of modern bees, dragonflies or diurnal crustaceans. The almost spherical receptor units are more separated and not as densely packed as in the hexagonal arrays of many modern compound eyes, but their internal structure very probably is almost identical. This 429-million-year-old trilobite already possessed a modern type of compound eye, and it is shown that the principles of vision in modern honey bee or dragon flies, as many crustaceans, is almost half a billion years old. Its excellent preservation expressly underlines the relevance and potentials of insights into the fossil record in understanding the evolution to functional principles to modern sensory systems of today.

## Material and methods

The photographs were taken with a Keyence digital-microscope (VHX-900F, VHZ-00R/0/T, VHZ-100R/W/T, VHZ-J20) at the Institute of Biology Education (Zoology), University of Cologne. The trilobite figured in this contribution is stored in the Geological Institute of the University of Cologne, GIK 191.
